# Prevalence and Antimicrobial Resistance of *Salmonella* Isolated From Dead-in-Shell Chicken Embryos in Shandong, China

**DOI:** 10.3389/fvets.2021.581946

**Published:** 2021-03-16

**Authors:** Xiaonan Zhao, Zijing Ju, Guisheng Wang, Jie Yang, Fangkun Wang, Hui Tang, Xiaomin Zhao, Shuhong Sun

**Affiliations:** ^1^Institute of Animal Science and Veterinary Medicine, Shandong Academy of Agricultural Sciences, Jinan, China; ^2^College of Animal Science and Technology, Shandong Agricultural University, Taian, China; ^3^Shandong Provincial Animal Disease Prevention and Control Center, Jinan, China

**Keywords:** chicken embryos, class 1 integrons, antimicrobial resistance, MLST, *Salmonella*

## Abstract

The present study was designed to explore the *Salmonella* prevalence and antimicrobial resistance characteristics in the context of chick mortality at hatching in China. Between December 2015 and August 2017, 1,288 dead-in-shell chicken embryos were collected from four breeder chicken hatcheries in Tai'an, Rizhao, Jining, and Heze, China. *Salmonella* isolates were successfully recovered from 6.7% of these embryos (86/1,288) and were evaluated for serotype, antimicrobial susceptibility, Class 1 integron prevalence, antimicrobial resistance gene expression, and multilocus sequence typing (MLST). *Salmonella* Thompson (37.2%), and *Salmonella* Infantis (32.6%) were the two most prevalent isolates in these chicken embryos, and 66.3% of isolates exhibited robust ampicillin resistance, while 55.8% of isolates exhibited multi-drug resistance (MDR). The majority of isolates harbored the *bla*_TEM_ gene (74.4%), with the *qnrS* gene also being highly prevalent (50.0%). In contrast, just 27.9% of these isolates carried Class 1 integrons. These 86 isolates were separated into four sequence types (STs), whereby ST26 (32.2%) was the most prevalent. Overall, these results suggested that *Salmonella* infections may be an important cause of chicken embryo mortality in China, and that efforts to support the appropriate use of antibiotics in managing poultry populations are essential.

## Introduction

*Salmonella* is an important foodborne pathogen that can cause serious illness in humans and animals (1). Over 2,600 *Salmonella* serovars have been detected to date (2), and these bacteria cause illness in roughly 1 million patients per year in the USA alone, leading to approximately $365 million in medical costs (3). Salmonellosis is also highly prevalent in China and is particularly common in elderly or immunocompromised individuals (4, 5). Most *Salmonella* infections occur as a consequence of the consumption of contaminated pork, poultry, or other foods, with poultry in particular being commonly identified as an important *Salmonella* reservoir species. A range of *Salmonella* serovars can infect poultry, causing significant morbidity and mortality and enabling horizontal transmission of these bacteria within flocks as well as vertical transmission of these bacteria to eggs, often resulting in embryonic mortality or death of newly hatched birds (6, 7). Prior work suggests that *Salmonella* infections are associated with 23.6% of dead breeder chicken embryos in Henan Province, China, with *Salmonella* Pullorum being the dominant serotype in this region (8). Similarly, *Salmonella* samples were isolated from 26.7% of dead-in-shell embryos in Jos, Central Nigeria, with *Salmonella* Hadar being dominant in this context (9). In order to control and prevent the spread of *Salmonella* through the food chain, it is vital that these sources of transmission and contamination be appropriately understood and managed.

Currently, *Salmonella* outbreaks are generally controlled *via* the application of antimicrobial agents. Widespread antibiotic application, however, has led to the emergence of antibiotic- and multidrug-resistant (MDR) *Salmonella* strains that can resist β-lactam and fluoroquinolone treatment, and that thus represent a major threat to global health (10–12). Such antimicrobial resistance can significantly increase treatment-related costs as well as rates of infection-related morbidity and mortality. The emergence of antibiotic-resistant *Salmonella* can occur in particular geographical regions and production sites, and may be confined to particular bacterial serotypes, emphasizing the importance of studying regional *Salmonella* epidemiology (13).

Bacterial genes associated with antibiotic resistance are commonly encoded by mobile genetic elements that can be transmitted between microbes, with DNA-based integrons being the primary mobile genetic elements responsible for the transmission of these genes *via* conjugation (14). The most common integrons in MDR *Salmonella* are class 1 integrons, which are also closely linked to resistance gene dissemination in a range of different pathogens (15).

Prior work has shown that the co-incubation of *Salmonella-*free and *Salmonella*-contaminated eggs can facilitate the horizontal transmission of these bacteria during hatching. After traversing the membrane, *Salmonella* can be extremely difficult to treat and generally further invades the egg whereupon it disrupts normal embryonic development (16, 17). Despite the critical importance of this pathogen, only a few studies have explored the prevalence of *Salmonella* in the context of chick mortality at hatching in Shandong, China (18). This study was therefore designed to assess *Salmonella* prevalence and antibiotic resistance characteristics in four breeder chicken hatcheries in this region in order to better understand the epidemiology of this foodborne pathogen.

## Materials and Methods

### Sample Collection

Between December 2015 and August 2017, 1,288 dead-in-shell chicken embryos were collected from four breeder chicken hatcheries in Tai'an, Rizhao, Jining, and Heze. The lungs, heart, liver, and trachea were taken from each embryo and pooled, transported on ice, and analyzed within 6 h of collection in a laboratory. A bacterial culture was conducted as discussed previously (19). Briefly, 100 mL of buffered peptone water (BPW; Hopebiol, Qingdao, China) was combined with samples, followed by an 18-h incubation at 37°C. Next, 1 mL of pre-enrichment culture was combined with 10 mL of selenite cysteine (SC; Hopebiol, Qingdao, China) broth for 24 h at 42°C. A loop was then used to streak a sample of this SC broth culture on xylose lysine tergitol 4 (XLT4; Hopebiol, Qingdao, China) agar plates, followed by incubation for 24 h at 37°C. A bacterial genome extraction kit (QIAGEN, Mississayga, Ontario, Canada) was then used based on provided directions to isolate bacterial DNA, and polymerase chain reaction (PCR) amplification of the *invA* gene was used to confirm the identity of presumed *Salmonella* colonies (20).

### *Salmonella* Serotyping

*Salmonella* isolate serotypes were established *via* the Kauffmann-White approach through slide agglutination using O and H antigen-specific sera (Tianrun Bio-Pharmaceutical, Ningbo, China) (21).

### Antimicrobial Susceptibility Testing

A Kirby-Bauer disk diffusion approach was used to evaluate *Salmonella* sensitivity to treatment with 12 different common antibiotics, as per the protocols of the Clinical and Laboratory Standards Institute (22). Antibiotics used for these tests included ampicillin (AMP), cephalosporin/acid (CAC), cefazolin (CFZ), chloramphenicol (CHL), ciprofloxacin (CIP), nalidixic acid (NA), polymyxin B (PB), fosfomycin (FFN), gentamicin (GEN), tetracycline (TET), streptomycin (STR), and sulfamethoxazole (SXT). As a control, the ATCC 25922 and ATCC 35218 *Escherichia coli* strains were utilized and purchased from Beina Biotechnology Co., Ltd. All *Salmonella* isolates that were found to resist more than three antibiotic classes were defined as being MDR strains.

### Class 1 Integrons and Antimicrobial Resistance Gene Detection

A bacterial genome extraction kit (QIAGEN) was used to isolate bacterial DNA, after which the *qnrA, qnrB, qnrC, qnrD, qnrS*, and *aac(6')Ib-cr* quinolone resistance genes were detected *via* PCR as detailed previously (23). Genes encoding β-lactamases, such as *bla*_TEM_, *bla*_PSE_, *bla*_CMY−2_, *bla*_SHV_, *bla*_DHA−1_, *bla*_OXA_, and *bla*_CTX−M_, were detected *via* PCR, as detailed previously (24, 25). Sequencing of all PCR products was then conducted. Class 1 integron gene cassettes were identified using primers and protocols discussed previously (26).

### MLST

Seven housekeeping genes were used for multilocus sequence typing (MLST) profiling (*aroC, dnaN, hemD, hisD, purE, sucA*, and *thrA*), as defined by the University of College Cork (http://mlst.ucc.ie/). The *Salmonella enterica* MLST database (http://mlst.warwick.ac.uk/mlst/dbs/Senterica) was used to assign STs to analyzed isolates.

Sequence-level relationships between *Salmonella* isolates were assessed by constructing an evolutionary phylogeny using MEGA6 *via* a maximum composite likelihood approach, with the topology of this phylogenetic tree being validated using 1,000 bootstrap replicates (27). To analyze resistance phenotypes and the relatedness of resistance gene expression profiles within this phylogenetic tree, the EvolView software package (http://www.evolgenius.info/evolview/#login) was used.

## Results

### *Salmonella* Prevalence

In total, we recovered *Salmonella* isolates from 6.7% of analyzed embryos (86/1,288), including 78 isolates from Tai'an (numbers 1–78) and 8 isolates from Rizhao (numbers 79–86). No isolate was recovered from Jining or Heze (Table 1).

**Table 1:** *Salmonella* prevalence in the context of chick mortality at hatching in Shandong.

**Locations**	**No. of samples**	**No. of positive samples**
Tai'an	313	78 (24.9%)
Rizhao	325	8 (2.5%)
Jining	325	0
Heze	325	0
Total	1,288	86 (6.7%)

Serotyping revealed four serotypes, including *S*. Thompson (*n* = 32), *S*. Infantis (*n* = 28), *S*. Enteritidis (*n* = 25), and *S*. Manhattan (*S*. Manhattan) (*n* = 1). *S*. Thompson (37.2%) and *S*. Infantis (32.6%) accounted for the majority of these isolates.

### Antimicrobial Susceptibility Testing

**Figure 1:**
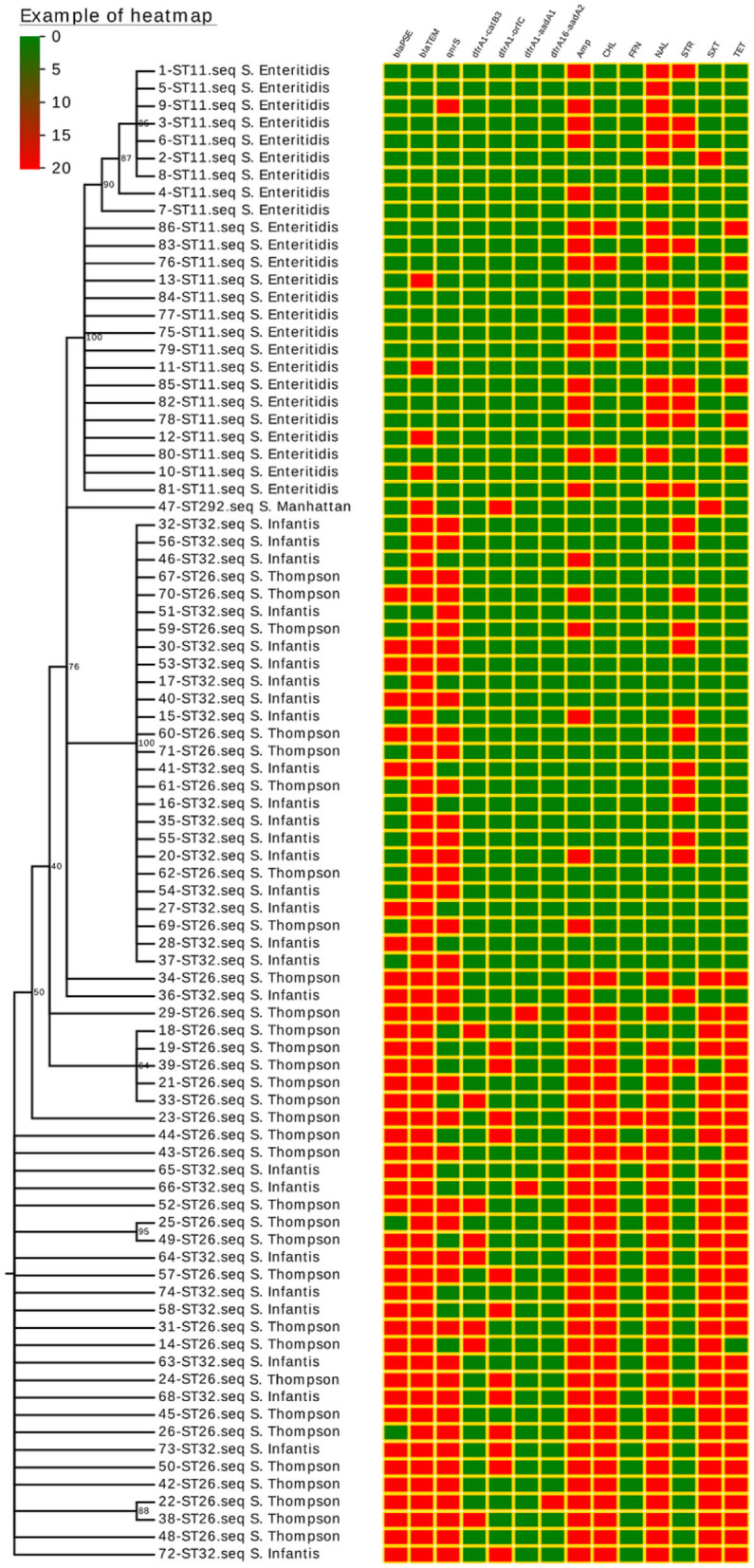
*Salmonella* isolate phylogenetic relationships, drug-resistance gene expression (*bla*_PSE_, *bla*_TEM_, and *qnrS*), class 1 integron structure (*dfrA1-catB3, dfrA1-orfC, dfrA1-aadA1*, and *dfrA1-aadA2*), and antimicrobial resistance (AMP, CHL, FFN, NAL, STR, SXT, and TET). Isolated strain numbers are given with ST designations and serotypes. Red and green squares correspond to the presence or absence of a given gene, respectively, or to the resistance or lack of resistance to a particular antibiotic, respectively.

These 86 *Salmonella* isolates were tested for resistance to 12 common antibiotics, revealing resistance rates as follows: ampicillin (66.3%), nalidixic acid (59.3%), tetracycline (47.7%), chloramphenicol (40.7%), sulfamethoxazole (38.4%), streptomycin (29.1%), and fosfomycin (2.3%). All *Salmonella* strains exhibited susceptibility or intermediate susceptibility to other tested antibiotics. Of these 86 isolates, 55.8% were classified as MDR isolates (Figure 1).

### Class 1 Integron and Antimicrobial Resistance Gene Detection

A single quinolone resistance gene (*qnrS*) was detected in these 86 *Salmonella* isolates, with this gene being carried by 50.0% of analyzed strains. Two different β-lactamase genes were found to be expressed in these isolates, with *bla*_TEM_ being the more common of the two (74.4%), followed by *bla*_PSE_ (46.5%). No isolates harbored the *bla*_CTX−M_, *bla*_CMY−2_, *bla*_SHV_, *bla*_OXA_, or *bla*_DHA−1_ genes (Figure 1).

Integrons were detected in 24 of these 86 *Salmonella* isolates (27.9%), and all *Salmonella* isolates harboring integrons were classified as MDR isolates with the exception of one isolate that was only resistant to two antibiotics. All detected *Salmonella* integrons encoded resistance gene cassettes, including the *dfrA1-orfC* (*n* = 13), *dfrA1-catB3* (*n* = 8), *dfrA1-aadA1* (*n* = 2), and *dfrA16-aadA2* (*n* = 1) cassettes (Figure 1).

### MLST

In an MLST analysis, these 86 *Salmonella* isolates were classified into four ST (Sequence Type) types, with ST26 being the most dominant (37.2%), followed by ST32 (32.6%), ST11 (29.1%), and ST292 (1.2%). These four STs were consistent with the observed serovars identified in this study, with ST26 corresponding to *S*. Thompson, ST32 to *S*. Infantis, ST11 to *S*. Enteritidis, and ST292 to *S*. Manhattan.

The majority of these ST strains were successfully grouped into a phylogenetic tree, which revealed no significant differences in drug-resistance gene expression patterns or drug-resistance spectra among strains within each ST (Figure 1).

## Discussion

In this study, we collected *Salmonella* isolates from 6.7% of analyzed chicken embryos, and the positive rate was similar to the eggs collected from poultry farms in Yangling (6.6%) (28) but lower than that from commercial chicken farms in China (25, 29). These differences in *Salmonella* isolation rates may be attributable to regional or seasonal differences, or to variations between studies with respect to the techniques used to collect samples. The relatively low isolation rate of *Salmonella* suggests that *Salmonella* is not the main cause of chicken embryo death, but it may be caused by other reasons, and further research is needed.

Serotyping is an effective approach to evaluating modes of transmission to develop strategies for preventing disease spread within poultry facilities (30). We found that *S*. Thompson, which is a member of *Salmonella* serogroup C1 commonly associated with disease in humans (31) and isolated in poultry and poultry eggs (8, 28, 32), was the most prevalent isolate in the present study. This is in contrast to studies in Shanghai and Sichuan that had identified *S*. Enteritidis as the most common serovar in commercial chicken farms (25, 33), while *S*. Indiana was found to be dominant in Shandong (34), and *S*. Weltevreden was dominant in Central Vietnam (35). Our study is the first to have reported the presence of *S*. Manhattan in Shandong, which is also found from broiler chickens in Kagoshima, Japan (36). Given that we observed clear overlap between the *Salmonella* serotypes isolated from chicken embryos and the strains known to cause human disease, this underscores the fact that *Salmonella* can be transmitted to humans through the consumption of contaminated food products (37).

Herein, we found that ampicillin and nalidixic acid were the most commonly resisted antibiotics, in line with findings from several other studies (38, 39), indicating that the use of these drugs may be widespread in laying hens. We also detected high rates of tetracycline resistance, consistent with the fact that this antibiotic is commonly used in the context of animal production (40). We found that the *Salmonella* isolates in the present study were largely sensitive to cephalosporin/acid, cefazolin, ciprofloxacin, polymyxin B, fosfomycin, and gentamicin, likely owing to the limited use of these antibiotics in the study area. We frequently detected MDR *Salmonella* isolates from dead-in-shell chicken embryos in the present study, consistent with findings from commercial chicken farms in China (41). In addition, *S*. Thompson showed a high MDR rate (24/32, 75.0%) in this study, which was different from other study that most of *S*. Indiana showed MDR (34). These MDR *Salmonella* isolates are of particular concern because they represent a major threat to public health if transmitted to humans through the food chain (42).

We found that the *qnrS* gene was expressed by the majority of isolates in the present study, in contrast to a prior study of commercial chicken farms in Shandong where this gene was not detected (44). We found that 22 *Salmonella* isolates harboring the *qnrS* gene were resistant to nalidixic acid, indicating that these strains may exhibit a chromosomal quinolone resistance-determining region point mutation. The *aac(6')Ib-cr* gene is a key mediator of bacterial resistance to ciprofloxacin and norfloxacin treatment (43). However, no bacteria harboring this *aac(6')Ib-cr* gene were detected in the present study, in contrast to the findings of a prior analysis of commercial Chinese chicken farms, which found this gene to be present in >90% of *Salmonella* isolates (44). The high prevalence of PMQR genes in *Salmonella* isolates underscores the importance of prudently utilizing fluoroquinolones in order to minimize the development of high-level fluoroquinolone resistance.

We found that *bla*_TEM_ was the most common β-lactamase gene expressed among isolates in the present study, followed by *bla*_PSE_, consistent with a similar report from commercial chicken farms in Shandong (34), although these results were inconsistent with those from a study of slaughterhouses and retail meat products in Sichuan, where the *bla*_OXA_ was the most common such gene, followed by *bla*_TEM_, *bla*_PSE_, and *bla*_CMY−2_ (25). We found that the majority of analyzed *Salmonella* isolates harboring *bla*_TEM_ and *bla*_PSE_ exhibited ampicillin resistance, indicating that β-lactamases may be the main mechanism in Gram-negative bacteria to overcome penicillin-derived antibiotics.

We found that 27.9% of our *Salmonella* isolates harbored integrons, consistent with a prior report from farm animals in Shandong (45), although this rate was higher than that reported for *Salmonella* isolates in the Netherlands (46). All but one of the *Salmonella* isolates harboring these integrons in the present study were classified as MDR isolates, consistent with a model wherein class 1 integrons are linked to the emergence of MDR in *Salmonella*. We additionally identified both *S*. Thompson and *S*. Infantis strains harboring these integrons.

An MLST approach led to the classification of these 86 *Salmonella* isolates into four STs, all of which have been previously linked to the incidence of human salmonellosis (31, 39). ST26 was the most prevalent ST in the present study, in contrast to the results of a prior study of breeder chicken flocks in nine Chinese provinces, which found ST92 to be the most prevalent in sample sites (18). We also observed a close relationship between STs and serovars. When a phylogenetic tree was used to assess relationships between ST genotypes and antibiotic resistance profiles, we observed marked similarities in drug-resistance characteristics for *Salmonella* isolates within each of these STs.

## Conclusion

In summary, we explored the characteristics of *Salmonella* infections in the context of chicken mortality at hatching in Shandong, China. We found the clinically important *S*. Thompson and *S*. Infantis serovars to be dominant among isolates recovered in the present analysis, and the majority of other isolates were also related to salmonellosis in humans. Overall, our data emphasize the importance of conducting antibiotic susceptibility testing when choosing appropriate antibiotics to treat *Salmonella* infections in order to minimize the risk of further facilitating the spread of drug-resistant strains of these dangerous bacteria.

## Data Availability Statement

The original contributions presented in the study are included in the article/supplementary material, further inquiries can be directed to the corresponding author/s.

## Author Contributions

XiaonZ, SS, and XiaomZ: data curation. SS and XiaomZ: formal analysis. SS: funding acquisition. XiaonZ, ZJ, and HT: investigation. ZJ, JY, GW, and FW: methodology. XiaonZ: writing. All authors contributed to the article and approved the submitted version.

## Conflict of Interest

The authors declare that the research was conducted in the absence of any commercial or financial relationships that could be construed as a potential conflict of interest.
